# The role of hypertension in the relationship between leisure screen time, physical activity and migraine: a 2-sample Mendelian randomization study

**DOI:** 10.1186/s10194-024-01820-4

**Published:** 2024-07-24

**Authors:** Quan Gan, Enfeng Song, Lily Zhang, Yanjie Zhou, Lintao Wang, Zhengming Shan, Jingjing Liang, Shanghua Fan, Songqing Pan, Kegang Cao, Zheman Xiao

**Affiliations:** 1 Department of Neurology, Renmin Hospital of Wuhan University, Wuhan, Hubei 430060 China; 2 Department of Traditional Chinese Medicine, Renmin Hospital of Wuhan University, Wuhan, Hubei 430060 China; 3 Department of Neurology, Dongzhimen Hospital, Beijing University of Chinese Medicine, Beijing, 100700 China; 4 Department of Encephalopathy in Traditional Chinese Medicine, Renmin Hospital of Wuhan University, Wuhan, Hubei 430060 China

**Keywords:** Lifestyle, Leisure screen time, Physical activity, Mendelian randomization, Migraine, Blood pressure

## Abstract

**Background:**

The relationship between lifestyle and migraine is complex, as it remains uncertain which specific lifestyle factors play the most prominent role in the development of migraine, or which modifiable metabolic traits serve as mediators in establishing causality.

**Methods:**

Independent genetic variants strongly associated with 20 lifestyle factors were selected as instrumental variables from corresponding genome-wide association studies (GWASs). Summary-level data for migraine were obtained from the FinnGen consortium (18,477 cases and 287,837 controls) as a discovery set and the GWAS meta-analysis data (26,052 cases and 487,214 controls) as a replication set. Estimates derived from the two datasets were combined using fixed-effects meta-analysis. Two-step univariable MR (UVMR) and multivariable Mendelian randomization (MVMR) analyses were conducted to evaluate 19 potential mediators of association and determine the proportions of these mediators.

**Results:**

The combined effect of inverse variance weighted revealed that a one standard deviation (SD) increase in genetically predicted Leisure screen time (LST) was associated with a 27.7% increase (95% CI: 1.14–1.44) in migraine risk, while Moderate or/and vigorous physical activity (MVPA) was associated with a 26.9% decrease (95% CI: 0.61–0.87) in migraine risk. The results of the mediation analysis indicated that out of the 19 modifiable metabolic risk factors examined, hypertension explains 24.81% of the relationship between LST and the risk of experiencing migraine. Furthermore, hypertension and diastolic blood pressure (DBP) partially weaken the association between MVPA and migraines, mediating 4.86% and 4.66% respectively.

**Conclusion:**

Our research findings indicated that both LST and MVPA in lifestyle have independent causal effects on migraine. Additionally, we have identified that hypertension and DBP play a mediating role in the causal pathway between these two factors and migraine.

**Supplementary Information:**

The online version contains supplementary material available at 10.1186/s10194-024-01820-4.

## Introduction

Migraine is a prevalent neurological disorder with a multifaceted etiology [1], remains the second leading cause of disability worldwide [2]. The identification and modification of lifestyle factors associated with migraine can inform research into the pathophysiology of migraine and facilitate the development of appropriate prevention and management strategies. A substantial body of evidence from both preclinical and prospective clinical studies indicates that unhealthy lifestyles, including physical inactivity, sleep disorders, and emotional disorders, smoking, alcohol consumption, may be associated with the development of migraine [3–6]. Nevertheless, the findings of extant observational studies are somewhat inconsistent.

Physical activity includes physical training, work labor, and daily living. As a modifiable lifestyle, physical training has been recommended as a preventative measure for migraine [7]. The increase in plasma β-endorphins after physical training, accompanied by the inhibition of substance P, may contribute to a reduction in pain pathway transmission [8]. Nevertheless, it has also been demonstrated that routine exercise may exacerbate the symptoms of migraine attacks, leading to a state of resistance to exercise [9]. This may be attributed to the fact that migraine, as a disabling neurological disorder, has an impact on patient’s ability to work and engage in social activities, which in turn reduces their level of physical activity [8]. It is therefore unclear whether migraine directly affects patients’ exercise or vice versa. This potential bidirectional causality is worthy of further investigation. Similarly, sleep and affective disorders are strongly associated with migraine, yet the causal link between the two remains controversial in different studies [10–12]. Alcohol, caffeine consumption, and smoking are the most common diet-related triggers associated with increased frequency of migraine attacks [13]. Some of the chemicals present in these lifestyle factors, including biogenic amines (such as histamine, tyramine, and phenylethylamine), caffeine, and nicotine, may play a role in the pathogenesis of migraine by stimulating neural pathways and triggering vascular reactions [14–16]. However, other studies have reached contradictory conclusions. Caffeine may attenuate pain perception and augment the analgesic efficacy of migraine headaches via its influence on adenosine receptors [17]. Conversely, studies have not found a correlation between coffee intake and the occurrence of migraine [18]. Additionally, with respect to the relationship between alcohol consumption and migraine, the extant literature indicates a negative correlation between alcohol consumption and migraine, which may be due to the tendency of migraineurs to avoid alcohol, rather than to the protective effect of alcohol itself in relieving migraine [14]. This indicates that the correlation between lifestyle factors and migraine may be more intricate than previously assumed.

The available evidence indicates that blood pressure fluctuations, metabolic and energy supply imbalances may be the key biological mechanisms in the pathogenesis of migraine [19]. Earlier research has indicated a higher prevalence of headaches among individuals with hypertension [20]. It has been shown that hypertension may affect the onset and development of migraine through several mechanisms. For example, hypertension may lead to abnormal vascular function, which in turn affects blood supply and neuromodulation in the brain, increasing the risk of migraine development. In addition, hypertension-induced sympathoexcitation and activation of the renin-angiotensin-aldosterone system may also interact with the pathophysiologic processes of migraine [21]. However, there are also epidemiological studies that suggest a negative correlation between headache occurrence and hypertension [22, 23]. The GWAS has demonstrated that migraine and blood pressure share genetic loci, the cross-trait correlation analyses unveiled potential common biological mechanisms between migraine and blood pressure regulation, involving vascular development, endothelial function, and neurogenic inflammation [24]. The association between blood pressure and migraine is inconsistent, a situation that may shed light on the implications of the existence of reverse causality of blood pressure on migraine in observational studies.

Furthermore, migraine is also closely associated with insulin resistance and metabolic syndrome [25, 26], which may lead to altered neuronal excitability, thereby increasing the susceptibility to cortical spreading depression (CSD) and triggering migraine attacks [27]. Furthermore, an imbalance of lipid metabolism plays an important role in the pathogenesis of migraine. It has been demonstrated that obesity increases the risk of migraine onset, attack frequency, and poorer prognosis. This may be related to the increased sensitivity of the trigeminal vascular system as a result of lipid dysregulation [28]. Additionally, calcitonin-related peptide (CGRP), the main pathogenic peptide associated with migraine, have been shown to play a key role in lipid metabolism and glucose homeostasis, thus affecting the pathogenesis of migraine [29, 30].

Furthermore, vitamins may play an important role in oxidative stress by acting as antioxidants, influencing the progression of migraine. For example, vitamin C can act as a scavenger of reactive oxygen species and ameliorate neuroinflammation in migraine [31]. Vitamin B6 deficiency can result in elevated homocysteine and an increased risk of neurovascular endothelial dysfunction, thus increasing the risk of migraine [32]. Furthermore, vitamin D supplementation has been found to prevent the development of migraine [33], potentially due to its anti-inflammatory, antioxidant, and neuroprotective effects [34]. An individual’s lifestyle is closely related to the body’s metabolic processes. We hypothesized that lifestyle factors may influence physiological indicators such as blood pressure, blood glucose, lipids, and vitamin levels, which in turn affect migraine risk. Determining whether these modifiable metabolic factors mediate lifestyle effects on migraine will provide a new theoretical basis for clinical practice and new avenues for migraine prevention and treatment [35]. Mendelian randomization (MR) is an epidemiological approach that utilizes genetic variation to infer causal relationships between biological factors and disease outcomes. By leveraging the effects of randomly assigned genotypes on phenotypes in natural settings, MR mimics the design of a naturally randomized controlled study, thereby mitigating confounding factors and the influence of reverse causality commonly encountered in observational studies. Multivariate MR (MVMR) extends the MR framework by enabling the incorporation of genetic variants associated with multiple potentially relevant exposures within the analysis, which enables the estimation of the independent impact of each exposure on the outcome while mitigating the influence of confounding bias. Consequently, MVMR can also be employed to estimate mediating effects [36].

This study employed a bidirectional two-sample MR (TSMR) analysis to investigate the independent causal relationships between 20 lifestyle behaviors and the susceptibility to migraine, while also investigating the potential mediating role of metabolic factors between lifestyle and migraine through mediation effect analysis. The aim was to elucidate the underlying biological mechanisms and provide novel evidence for the prevention and therapeutic management of migraine.

## Methods

### Study design

**Fig. 1:**
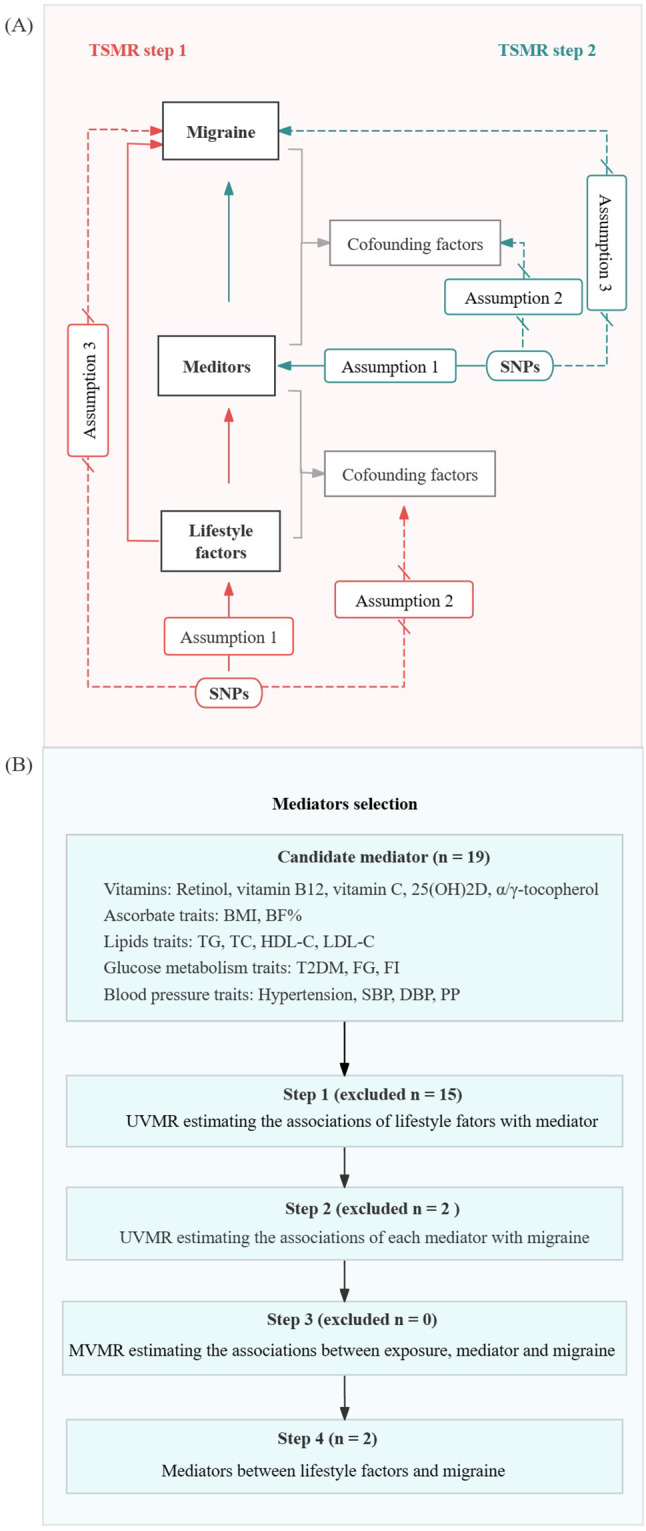
Diagram of the study design. (**A**) Study design; (**B**) Selection process for mediating variables. *Abbreviations* BMI, body mass index; BF%, body fat percentage; TG, total triglyceride; TC, total cholesterol; HDL, high-density lipoprotein; LDL, low-density lipoprotein; T2DM, type 2 diabetes; FG, fasting glucose; FI, fasting insulin; SBP, systolic blood pressure; DBP, diastolic blood pressure; and PP, pulse pressure

The present MR study included a total of 39 categories of modifiable factors (20 lifestyle and 19 metabolic traits). The study comprised two stages of analyses, as depicted in Fig. 1A for the study design. In the first stage, we evaluated the independent causal effects of each lifestyle category on migraine using TSMR and MVMR. Subsequently, in phase 2, we examined potential mediators for the associations between lifestyle and migraine and conducted a mediation analysis to determine and quantify the impact of the mediator’s effect on the association between lifestyle on migraine (Fig. 1B).

### Data sources of lifestyle factors, mediators, and migraine

For the MR analyses, we obtained GWAS datasets from both the Open GWAS and GWAS catalogs. We screened datasets that provided complete summary statistics on European ancestry for each variable. Detailed information on the GWAS datasets, including the number of participants and adjusted covariates, is presented in Table S1. All studies included in the cited GWASs had received approval from a relevant review board, and all participants had provided consent forms. Our study adhered to the scope outlined in the original ethics committee approval.

### Lifestyle factors

**Fig. 2:**
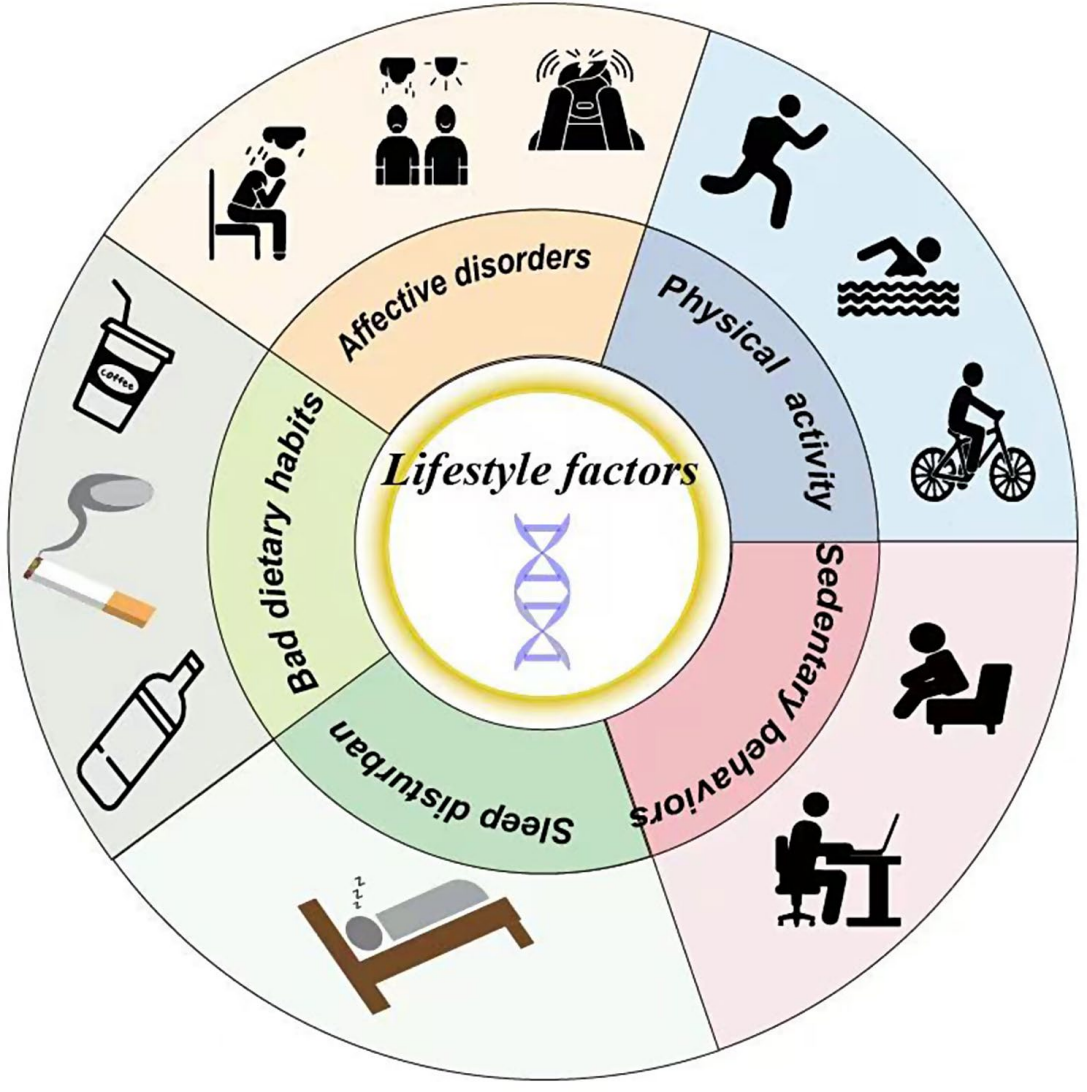
Lifestyle variables examined in the study

Lifestyle variables included physical activity, sedentary behaviors, sleep disturbances, smoking, alcohol consumption, coffee intake, and affective disorders (Fig. 2).

### Data sources on physical activity (PA) and sedentary behavior

Genetic instruments for PA and sedentary traits were derived from the latest GWAS meta-analysis dataset, including up to 661,399 European individuals from 53 studies with questionnaire-based data [37]. PA was measured using self-reported moderate-to-vigorous physical activity (MVPA). The sedentary behavior phenotype included three self-reported measures: sedentary behavior at work, sedentary commuting, and leisure screen time (LST). PA and sedentary behavior were dichotomized based on self-reported outcomes. To ensure consistency between studies, PA was categorized as “active” and “inactive”. Specifically, subjects were categorized as physically active if they were physically active for an average of 20 min or more per week, and inactive if they did not. The term “MVPA” encompasses aerobic exercise (e.g., jogging, running, cycling, skiing, ball games, etc.) and fitness exercise. It excludes activities that are primarily occupational, such as shoveling or weightlifting, as well as light leisure activities, such as walking or gardening. The definition of sedentary behavior was derived from the subjects’ self-reported sedentary status at work and during commuting time. Accordingly, subjects were classified into two categories: sedentary and non-sedentary behaviors, and LST was quantified as hours per day.

### Data sources on sleep disturbance

Genetic instruments for sleep disturbances were obtained from the Sleep Disorder Knowledge Portal (SDKP), and sleep phenotypes in this study included sleep chronotype, sleep duration, long/short sleep duration, and insomnia [38–40]. Specifically, chronotype data were derived from a single self-report question: “What type of sleeper do you consider yourself to be?” The responses “Definitely a ‘morning’ person” and “More of a ‘morning’ person than a ‘night’ person” were categorized as “morning type”, “More of an ‘evening’ person than ‘morning’” and “Definitely an ‘evening’ person” were categorized as “evening type”, and other responses such as “don’t know or “don’t want to answer” were excluded [38]. Participants reported sleep duration as a continuous variable based on self-reported habitual sleep duration per day, and was also divided into short sleep (6 hours or less), normal sleep (7 or 8 hours), and long sleep (9 hours or more). Participants with extreme sleep duration less than 3 hours or greater than 18 hours, uncertainty, and use of any sleep medication were excluded [39]. Cases of insomnia were determined from self-report to the question, “Do you have trouble falling asleep at night or do you wake up in the middle of the night? " with responses never/rarely, sometimes, usually, prefer not to answer. Subjects who responded “prefer not to answer” were set to missing. One in which insomnia symptoms were dichotomized into controls (“never/rarely”) and cases with any symptoms (“sometimes” and “usually”) [40].

### Data sources on bad dietary behaviors

GWAS summary statistics data on smoking and alcohol behavior were obtained from the GWAS & Alcohol and Nicotine Sequencing Consortium (GSCAN). Four sets of genetic instruments were evaluated with smoking behavior, encompassing the age at which individuals began smoking regularly, the quantity of cigarettes consumed daily, the regularity in smoking, and the act of quitting smoking. Smoking initiation phenotypes included age of initiation of regular smoking and a binary phenotype indicating whether an individual had ever smoked regularly. Heaviness of smoking was measured with cigarettes per day. Smoking cessation was a binary variable contrasting current versus former smokers. Smoking phenotypes do not include information on pipe/cigar/chewing or other non-cigarette forms of tobacco use. Alcohol consumption was defined as the average number of drinks a participant reported drinking each week, aggregated across all types of alcohol [41]. The genetic variables related to coffee intake were obtained from the UK Biobank (UKB) cohort. Participants completed a 24-hour recall questionnaire, and their coffee intake was determined based on the average consumption reported in at least two dietary recalls, including decaffeinated coffee [42].

### Data sources on affective disorders

GWAS data on Sensitivity to environmental stress and adversity (SESA) cluster were derived from the UKB, and everyone’s phenotype was according to three items of the Eysenck Personality Questionnaire-Revised Short Version (EPQ-RS), including “Are your feelings easily hurt?“, “Do you worry too long after an embarrassing experience?” and “Are you often troubled by feelings of guilt?” Participants with information on all these three questions were included [43].

Genetic instruments for the remaining subclusters of affective disorders selected, including neuroticism, depression, depressed affect, and worry, were derived from a meta-analysis of GWAS data from the UKB cohort, the Genetics of Personality Consortium (GPC) cohort, and the Psychiatric Genetics Consortium (PGC), as reported by Nagel et al. [44]. The UKB measured neuroticism using a 12-item EEPQ-RS [45], and a weighted mean score was calculated as a total score. In the GPC cohort, neuroticism was measured using the 12 five-point scale items of the NEO Five-Factor Personality Inventory (NEO-FFI), and a weighted mean score was also calculated [46]. Depression was assessed in the UKB cohort via two questionnaire items: “How often have you felt down, depressed, or hopeless in the past two weeks?” and “In the past two weeks, how often have you lacked interest or pleasure in doing things?” Response options for each item were “Not at all” (1 point), “A few days” (2 points), “More than half the days” (3 points), and “Almost every day” (4 points). The scores from the two questions were summed to form a “depression index,” which was standardized to have a mean of 0 and a variance of 1. This index was employed to quantify the degree of depression. In the GPC cohort, the depression measure was dichotomous, and depression was diagnosed if there were at least two diagnoses of depression according to the International Classification of Diseases-9th (ICD-9) classification. The PGC categorizes individuals with a lifetime diagnosis of major depressive disorder as cases, which meet the criteria in the Diagnostic and Statistical Manual of Mental Disorders (DSM).

The sum of scores on four EPQ-RS items (i.e., “Do you often feel lonely? “, “Do you ever feel “just miserable” for no reason? “) was used to obtain scores for the cluster depressed affect. Similarly, the sum of scores on four other EPQ-RS items (i.e., “Does your mood often go up and down? “, and “Do you often feel “fed up”?“. The GWAS for worry cluster was obtained by summing the score on these four items, “Are you a worried? “, “Do you suffer from nerves? “, “Would you call yourself a nervous person? “, and “Would you call yourself tense or highly strung”) was used to obtain scores for the cluster worry.

### Data sources on mediators

We identified 19 candidate mediator variables that may lie within the causal pathway between lifestyle and migraine. The detailed information of epidemiological evidence for the relationship between the studied lifestyle factors and mediators with migraine is provided in Table S2, and Table S3 provides detailed information on GWAS of studied mediators, including vitamin traits (retinol, vitamin B12, vitamin C, 25-(OH)2D, α-tocopherol, γ- tocopherol) [47–50], adiposity traits (body mass index [BMI] and body fat percentage [BF%]) [51, 52], lipids traits (total triglyceride [TG], total cholesterol [TC], high-density lipoprotein - cholesterol [HDL-C], low-density lipoprotein - cholesterol [LDL-C]) [53], glucose metabolism-related traits (fasting glucose, fasting insulin, type 2 diabetes) [54, 55], and blood pressure traits (hypertension, systolic blood pressure [SBP], diastolic blood pressure [DBP], and pulse pressure [PP]) [55, 56].

### Data sources on migraine

A summary-level data set on migraine was collected from two large studies. The data from the Discovery Set study were derived from the FinnGen database and included 18,477 individuals with migraine and 287,837 controls [57]. The cases of migraine were defined according to the International Classification of Diseases-10th (ICD-10) classification code G43 and ICD-9 code 346. The dataset excluded individuals of indeterminate sex, high genotypic deletion rate (> 5%), excessive heterozygosity (± 4 SDs) and individuals of non-Finnish ancestry, and the association test was adjusted for age, sex, genetic ancestry principal components (PCs), and genotyping batches. The largest GWAS dataset of migraine European samples to date from the Genetic Epidemiology Research in Adult Health and Aging (GERA) cohort, the UKB cohort, and pooled data from the International Headache Genetics Consortium (IHGC) of Gormley et al., comprising a total of 26,052 cases, 487,214 controls for replication-stage MR analyses [58]. Migraine cases were defined by code G43 in ICD-10 and code 346 in ICD-9, and some of the UKB cohort cases were self-reported by patients. The UKB data in the replication set excluded individuals of non-European ancestry, closely related individuals (or at least one of a related pair of individuals), individuals with sex chromosome abnormalities, and individuals who withdrew consent from the UKB study. The replication set was adjusted for age, sex, and ancestry PCs.

### Genetic instrument selection

Independent single nucleotide polymorphisms (SNPs) associated with these factors were selected from the corresponding GWASs using a significance threshold of *P* < 5 × 10^− 8^. Genetic instrument selection for the MVMR analysis followed the same criteria. The instrumental variables (IVs) were clumped based on the linkage disequilibrium (LD) structure from the 1000 Genomes Project, with a threshold of r^2^ < 0.001 and a clump window of 10,000 kb to remove any correlated variants [59].

### Statistical analysis

#### TSMR and MVMR analyses

We conducted a two-sample bidirectional MR study using genetic proxies to evaluate the causal relationship between modifiable lifestyle and migraine. A forward MR analysis was conducted to assess the impact of genetic predisposition to lifestyle factors on the risk of developing migraine. Additionally, a reverse MR analysis was employed to explore the potential influence of genetic susceptibility to migraines on these lifestyle factors. Subsequent MVMR analyses were performed to evaluate the direct causal impact of a specific lifestyle factor on migraine, while accounting for the influence of other lifestyle factors through adjustment. All MR analyses met three essential assumptions: (i) The instrumental variables exhibited a strong correlation with the exposure variable, satisfying the hypothesis of association; (ii) The instrumental variables were independent of the confounding factors, satisfying the independence assumption; (iii) The instrumental variables were not associated with outcome variables but only with outcome variables through the exposure variables, satisfying the independence assumption, the three hypotheses to be satisfied in this study are depicted in Fig. 1A. The inverse variance weighted (IVW) method was used as our main MR analytical approach. To augment the statistical robustness of the analysis, we combined the IVW estimates of the discovery and replication sets through fixed-effects meta-analysis [60].

#### Mediation MR analyses

To screen for mediators that regulate the causal pathway between lifestyle and migraine, the following criteria should be applied:

1. The effect of lifestyle on the mediator should be unidirectional.
2. There should always be a causal relationship between the mediator and migraine with or without lifestyle modification.
3. The direction of the lifestyle-to-mediator effect as well as the mediator-to-migraine effect should be consistent.

The flow chart of the process of candidate media screening is shown in Fig. 1B. The TSMR was used to estimate the causal effect of lifestyle factor on migraine in the two GWAS datasets, and the effects were combined to generate a total effect β. Subsequently, the TSMR was conducted to estimate the effect size β1 of lifestyle on the mediator, and ultimately estimated the effect size β2 of the mediator-to-migraine effect after adjusting for lifestyle using multivariate MR. The indirect effect was calculated by multiplying the effect sizes of the two steps (β1 × β2). The mediating effect was calculated using the formula indirect/total effect (β1 × β2/β), and standard errors (se) were inferred using the Delta method utilizing the effect estimates obtained from the two-sample MR analysis.

#### MR sensitivity analyses

In sensitivity analyses, other MR methods such as MR-Egger, Weighted median were performed to correct any potential violations of the assumptions. To check the consistency of results and horizontal pleiotropy, sensitivity analyses were performed using weighted median, MR-Egger regression, and MR-Pleiotropy RESidual Sum and Outlier (MR-PRESSO). The weighted median method produces consistent causal estimates if more than half of the instrumental variables used are valid. MR-Egger regression detects horizontal pleiotropy by the p-value of its intercept test. MR-PRESSO to identify horizontal pleiotropic and identify and discard influential outlier predictors from the IVW test. Additionally, if any pleiotropic SNP was found through the PhennoScanner analysis for causality associated pairs, we removed each possible variant separately and conducted the primary method of IVW again. Cochran Q analysis to assess heterogeneity and considered the fixed-effects IVW approach as the main approach if *P*-values were higher than 0.05 without evidence of heterogeneity. The random-effects IVW approach was utilized if there was substantial heterogeneity (*P* < 0.05). The leave-one-out sensitivity analysis was used to assess the effect of individual SNPs on causal estimates by removing each SNP alternatively. The F statistic was calculated to assess the strength of IVs, an F-statistic of > 10 indicated that there was no obvious weak instrument bias.

In TSMR analyses associated with genetic susceptibility to migraine, correlations with *p*-values less than 0.001 (0.05/39 exposures) were deemed a significant association, and the association with the *P*-value ≥ 0.001 and ≤ 0.05 were regarded as a suggestive association. The *P*-value for TSMR associated with LST was set at 0.003 (0.05/19 exposures). IVW estimates were considered causally related only if they were in the same direction and statistically significant as at least one of the sensitivity analyses and showed no evidence of pleiotropy. All statistical tests were two-sided and performed using the TwoSampleMR, Mendelian Randomization and MRPRESSO packages in RStudio 6.0.421.

## Results

### Lifestyle factors and migraine

**Fig. 3:**
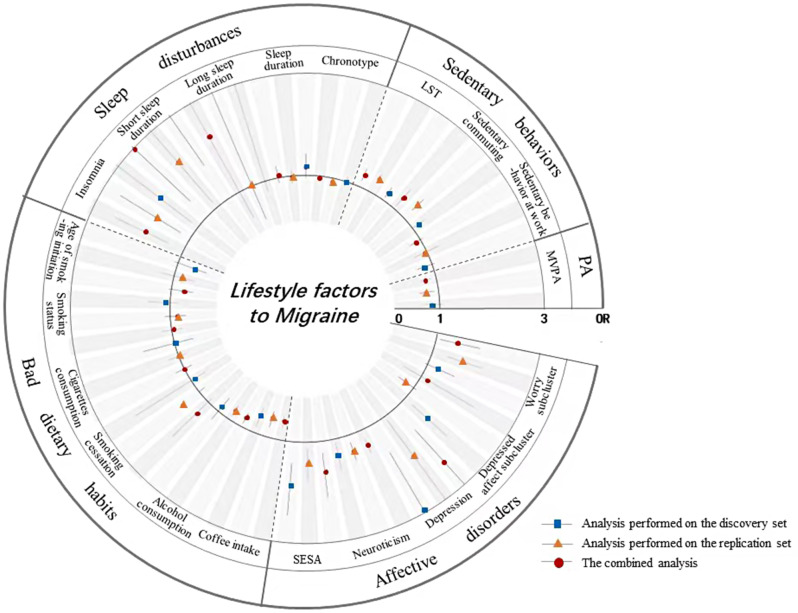
UVMR estimates of the casual association of each lifestyle factors with migraine. Blue, yellow, and red colors represent the causal effects of the genetically predicted lifestyle with migraine for the discovery set, replication set, and merged set, respectively, estimated using the IVW statistical method. Abbreviations: PA, physical activity; MVPA, moderate or/and vigorous physical activity; LST, leisure screen time; SESA, sensitivity to environmental stress and adversity

Among the 20 lifestyle phenotypes, it was found that genetic susceptibility of five phenotypes was associated with an increased risk of migraine, while two phenotypes were associated with a decreased risk of migraine (Fig. 3). The combined of IVW analysis indicated that the genetically predicted depression (OR, 2.63; 95%CI, 1.66–4.2), neuroticism (OR, 1.36; 95%CI, 1.19–1.56), SESA (OR, 1.69; 95%CI, 1.26–2.27), insomnia (OR, 1.94; 95%CI, 1.40–2.70), and LST (OR, 1.28; 95%CI, 1.14–1.44) were all associated with an increased risk of migraine. However, a genetically predicted 50% increase of coffee intake was found to be associated with a 43.3% reduction in the risk of migraine (OR, 0.57; 95%CI, 0.44–0.73), MVPA was linked to a 26.9% decrease in the risk of migraine (OR, 0.73; 95%CI, 0.61–0.87), the comprehensive findings of discovery and validation analyses are shown in Table S4. In the reverse MR analysis, an observed correlation was found between genetic predisposition to migraine and a decrease in coffee consumption, as well as an evaluated likelihood of morning chronotype and insomnia (Table S5). It is worth noting that all utilized genetic instrumental variables did not exhibit pleiotropy with the outcome, although certain genetic instrumental variables displayed heterogeneity (Table S4 and Table S5).

**Fig. 4:**
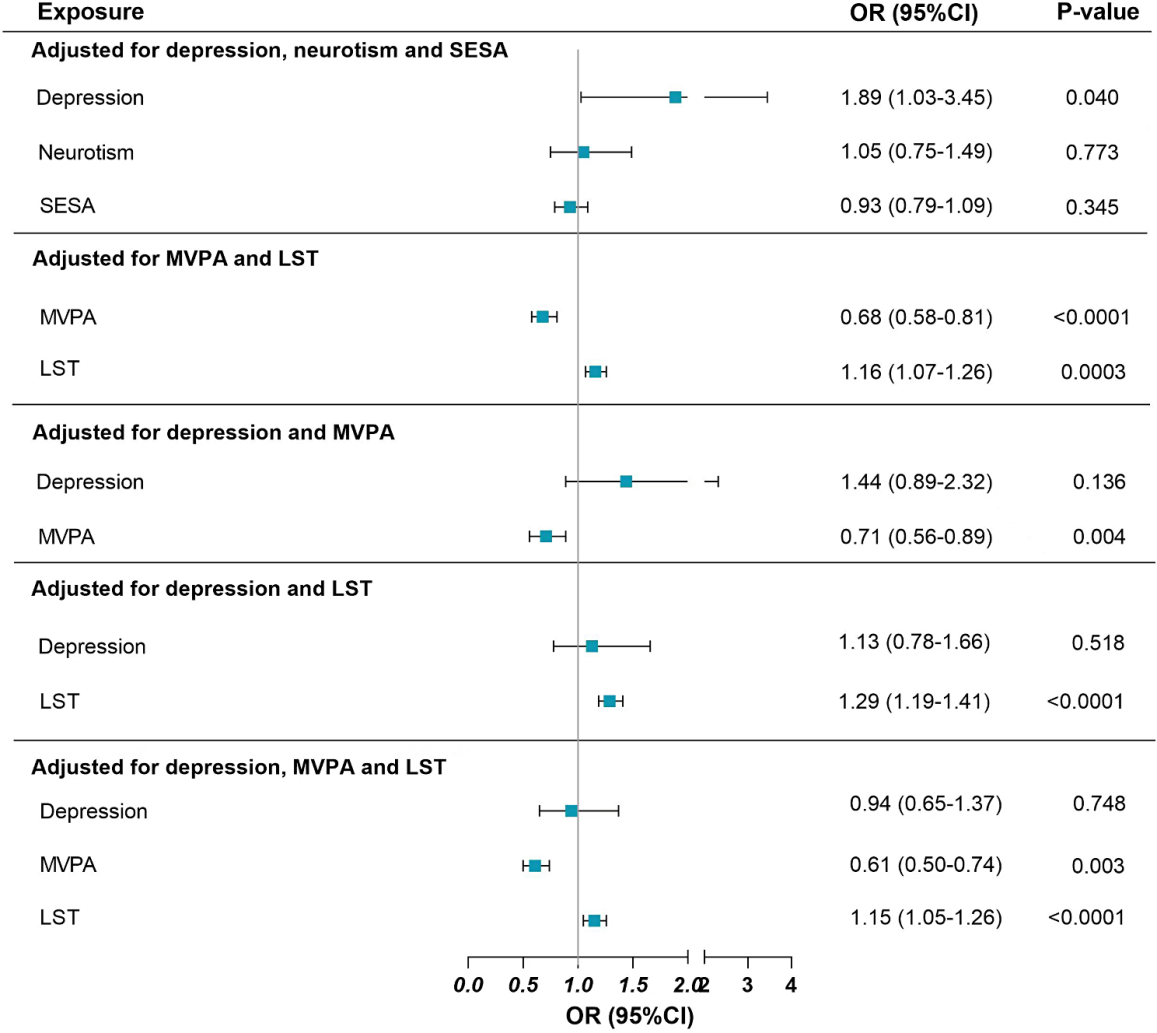
The IVW analysis method in the MVMR estimated the relationship between genetically predicted lifestyle factors and migraine after adjusting for other factors

Subsequently, the MVMR approach was employed to concurrently assess the causal relationship between each phenotype and migraine. Following adjustment for genetic instrumental variables pertaining to two other mental disorders, including neuroticism and SESA, the causal effect of depression on migraine remained (OR, 1.89; 95%CI, 1.03–3.45). Furthermore, upon accounting for PA and sedentary behavior, both genetically predicted MVPA and LST exhibited causal associations with migraine (OR, 0.68; 95%CI, 0.58–0.81 for MVPA, OR, 1.16; 95%CI, 1.07–1.26 for LST, respectively). However, upon controlling for all relevant variables that exerted a causal influence on migraine, the causal association of depression with migraine was no longer statistically significant. Instead, only MVPA and LST exhibited distinct and independent causal effects on genetical prediction of migraine, as illustrated in Fig. 4.

### LST, MVPA and mediators

Bidirectional mediation analysis was employed to examine the potential impacts of LST or MVPA in relation to mediating factors. The forward MR findings revealed significant correlations between BMI and hypertension with LST, as well as between hypertension, SBP, and DBP with MVPA (Table S6). A 1-standard deviation increase in LST was found to be associated with a significant increase in BMI of 0.18 kg/m² (95% CI, 1.13–1.23; *P* = 7.96 e-14), as well as a substantial 27.9% increase in the risk of hypertension (95% CI, 1.20–1.36; *P* = 7.95e-15). Furthermore, engaging in MVPA was found to be associated with the 34% decrease in hypertension risk (95% CI, 0.56–0.78; *P* = 4.17e-07), a significant 94% decrease in SBP (95% CI, 0.002-0.20; *P* = 4.99e-06), and an 80% decrease in DBP (95% CI, 0.11–0.34; *P* = 2.62e-09). The results of the reverse MR analysis revealed a positive relationship between BMI and the genetic prediction of the likelihood of LST, while a negative relationship was observed between BMI and MVPA (Table S7). No evidence was found for directional pleiotropy, as indicated by the non-significant MR-Egger intercepts. Additionally, no relationships were found between the other mediators examined, including BF%, T2DM, FG, FI, TG, TC, HDL, LDL, PP, with LST or MVPA.

### Mediating effects of mediators in the association between MVPA, LST and migraine

**Fig. 5:**
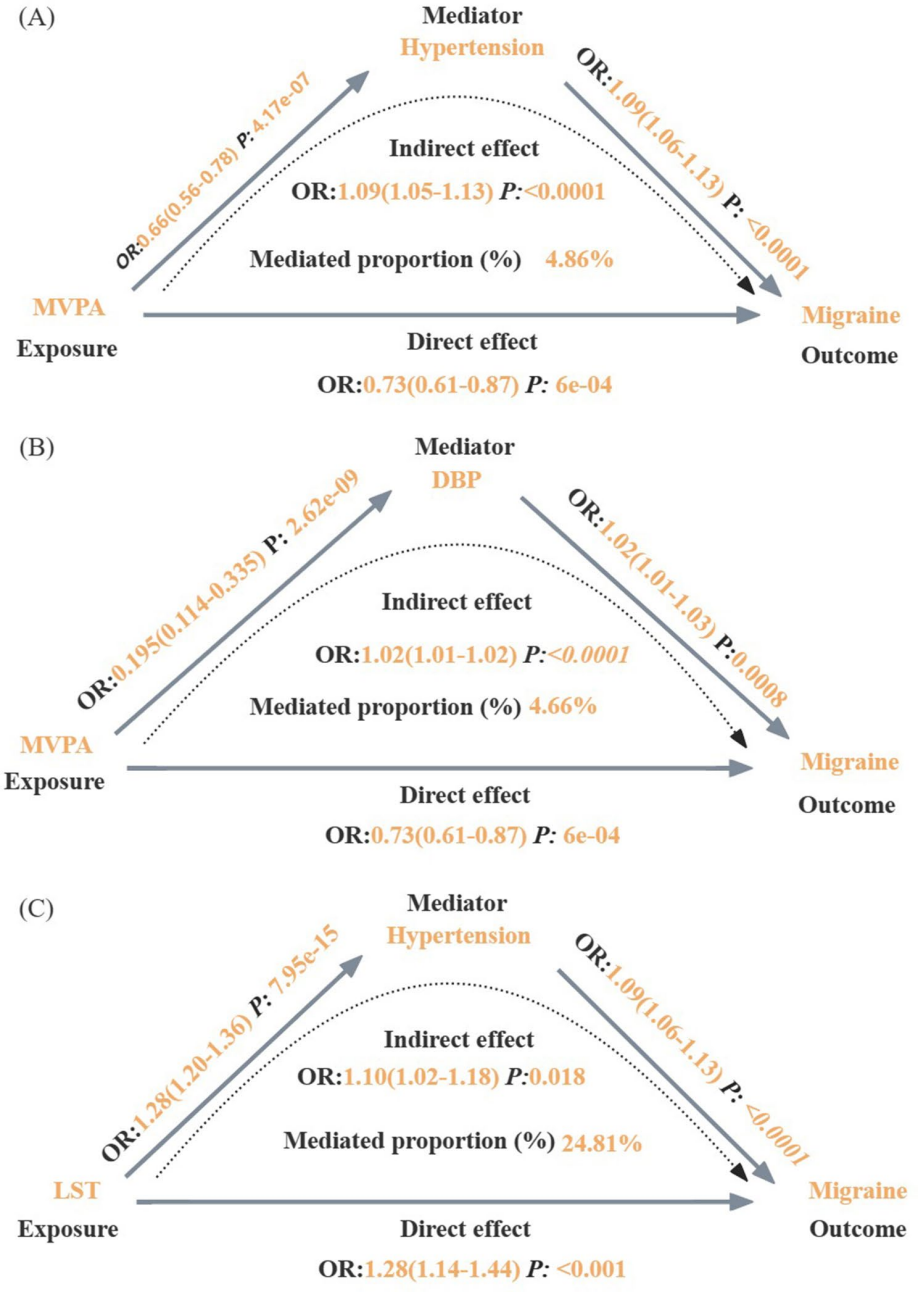
MR estimates of proportions mediated by mediators in the causal relationship between MVPA/LST and migraine. (**A**) Causal effects between MVPA, hypertension and migraine. (**B**) Causal effects between MVPA, DBP and migraine. (C) Causal effects between LST, hypertension and migraine

Finally, we utilized MVMR and mediation analyses to examine the potential impact of blood pressure variables on the relationship between LST or MVPA and migraine. The UVMR findings revealed that genetically predicted hypertension was linked to a 9% higher risk of migraine (OR, 1.09; 95%CI, 1.06–1.13), and that genetically predicted migraine risk increased by 1.8% for each 1-mm Hg increase in DBP (OR, 1.02; 95%CI, 1.01–1.03), without any evidence of directional pleiotropy. However, no significant association was observed between genetic susceptibility to SBP and migraine (Table S8). Reverse MR analysis did not find a causal association between genetically predicted migraine and blood pressure variables (Table S9). Out of the 19 candidate mediators, only two modifiable risk factors that met the screening criteria were included in the mediated MR analysis of MVPA or LST with migraine. Upon adjusting for genetic susceptibility to hypertension, the associations between both MVPA and LST and migraine were attenuated, with genetic susceptibility to hypertension mediated 4.86% and 24.81% of the effects of MVPA and LST on migraine risk, respectively. Furthermore, the genetic prediction of DBP accounted for 4.66% of the effect of MVPA to migraine (Fig. 5).

## Discussion

Migraine is a debilitating disease that severely affects the quality of life and work ability of patients, with high rates of social morbidity and disability [61]. Thus, it is crucial to identify effective prevention strategies for migraine to enhance public health. Recent research indicates that embracing a healthy lifestyle may diminish the likelihood of experiencing migraine [7]. Nonetheless, given that the available evidence primarily stems from observational studies, the causality of the association between lifestyle and migraines remains uncertain. Consequently, while advising against unfavorable lifestyles may prove advantageous for overall health, its specific impact on migraines remains inconclusive. We conducted bidirectional univariable and multivariable MR analyses using extensive genome-wide association discovery set and replication set datasets to exam potential causal associations between lifestyle, metabolic traits, and the likelihood of developing migraine. Our findings revealed that engaging in MVPA and coffee consumption were casually linked to a reduced risk of migraine. Conversely, factors such as LST, insomnia, depression, neuroticism, and SESA were identified as potential detrimental contributors to migraine susceptibility. There was evidence to suggest that migraine could alter sleep patterns to favor morning chronotypes and decrease alcohol consumption. Furthermore, a reciprocal causal relationship between migraine and insomnia was observed. Our study utilizing MR provided novel evidence for the causal connection between MVPA and LST on migraine, even after accounting for other aspects of lifestyle. Intriguingly, the enduring causal impact of other lifestyle factors on migraine was not evident after adjusting for confounding variables, suggesting that their effects on migraine is largely influenced by additional lifestyle factors.

The term MVPA in this study includes both aerobic exercise and strength training. Several clinical trials have confirmed the efficacy of exercise interventions for the treatment of migraineur [62, 63]. Whether it is aerobic exercise (high-intensity aerobic, moderate-intensity aerobic), strength training, or even multimodal exercise training, it is effective in reducing migraine burden [64, 65]. The impact of exercise on the clinical progression of migraine has been explored through various explanatory mechanisms. On the one hand, studies have shown that exercise reduces pain perception by activating the endogenous cannabinoid system and the endogenous opioid system [66]. Concurrently, exercise can also increase the release of various neurotrophic factors, including brain-derived neurotrophic factor (BDNF), insulin-like growth factor (IGF-1), and vascular endothelial growth factor (VEGF), which promote neuronal plasticity and angiogenesis, as well as improve cerebral blood flow and perfusion [64, 67]. However, the above mechanisms remain speculative. Our results revealed that MVPA exerted a protective influence on migraine risk by mitigating the likelihood of developing hypertension. Persistent hypertension leads to fibrosis of the blood vessel wall and damage to the vascular endothelium. Several studies have demonstrated that moderate- to high-intensity physical training has a beneficial effect on vascular endothelial function [68, 69]. Meanwhile, the cross-trait correlation analyses have unveiled potential common biological mechanisms between migraine and blood pressure regulation, involving vascular development, endothelial function, and neurogenic inflammation [70]. This suggests that vascular endothelial function may play a critically important role in the triad between exercise, hypertension, and migraine. Specifically, during exercise, increased blood flow shear stress stimulates endothelial cells and promotes the upregulation of endothelial nitric oxide synthase (eNOS) and VEGF expression [71]. These changes contribute to the enhancement of endothelium-dependent vasodilatation while inducing neovascularization, thereby improving systemic vascular endothelial function [68]. This positive modulation of endothelial function contributes to the eventual reduction of blood pressure levels. Conversely, sustained hypertension can additionally impair vascular endothelial function, resulting in a reduction in endothelium-dependent vasodilation, which in turn exacerbates vascular wall fibrosis and inflammation [72]. Endothelial dysfunction-induced vasoconstriction, microcirculatory disorders, and neurogenic inflammation may represent a pivotal mechanism in the pathogenesis of migraine attacks [73]. Engaging in MVPA during leisure time has been demonstrated to reduce the burden of migraine headaches when compared to the effects of physical activity undertaken for commuting or work [8]. As a lifestyle-based intervention, regular exercise has been demonstrated to not only reduce the frequency of migraine attacks but also to exert a beneficial effect on blood pressure control.

The amount of research on the relationship between screen time and headache is remarkably small, but the existing studies have indicated that in healthy children, adolescents, and young adults, headaches are more prevalent with increased screen time [74, 75]. This is consistent with our findings that recreational screen time increases the risk of migraine after controlling for other lifestyles. Two potential hypotheses have been proposed to explain how screen time interacts with migraine pathophysiology. The first hypothesis suggests that the brightness or frequency of light in the screen band may directly trigger a migraine attack. The second hypothesis proposes that increased screen time exposure may lower the threshold for migraine cascade and then be induced by other factors [76]. Studies have shown that increased screen time raises body fat levels and the risk of developing metabolic syndrome [77, 78], including some of the metabolic triggering factors that result in migraine attacks, such as insulin resistance, high blood pressure, and dyslipidemia [79, 80]. However, our study did not find an association between lipid and glucose metabolism and genetic susceptibility to migraine. We revealed that LST increases the risk of developing hypertension, which in turn increases susceptibility to migraine. Previous studies have indicated that prolonged television viewing was associated with higher SBP and DBP, whereas increased physical activity was primarily associated with lower DBP [81]. Consequently, interventions aimed at reducing blood pressure levels among individuals who are physically inactive or spend prolonged time in front of screens may effectively diminish the occurrence of migraine.

Surprisingly, in our study, certain mediators supported by observational studies performed no mediating role in the relationship between lifestyle to migraine [82, 83]. Our TSMR findings showed a suggestive causal association between some indices of glucose and lipid metabolism with LST or MVPA. Specifically, LST showed a potential causal association with triglycerides, LDL, and fasting insulin, while MVPA showed a potential causal association with HDL and 25(OH)D. Additionally, there was bidirectional causality existed between genetically predicted BMI and LST, hence, it was excluded from our mediation analyses. Significant causal links were discovered between MVPA and type 2 diabetes, but not between type 2 diabetes and migraine, suggesting that the significant associations observed in observational studies may be partially influenced by residual confounders.

Our research provides a comprehensive analysis of the causal connections between different lifestyles and migraines. Unlike prior MR studies that solely investigated the relationships between a singular lifestyle factor and migraine, we employed MVMR analysis to independently evaluate the effects of each variable and ascertain the causal mediators that underlie their respective pathways. Our study on migraine utilized two GWAS data sets. The FinnGen study was chosen for its minimal overlap with exposure or mediator GWAS, thereby minimizing false-positive results. Additionally, the meta dataset of GWAS from Choquet et al. was employed to improve the statistical power for replicating and validating the findings of the FinnGen study, given its substantial sample size. The IVW estimates showed consistency with the results obtained from multiple sensitivity analyses. Moreover, our study rigorously established screening criteria for mediators to mitigate the influence of reverse causation between variables, thus providing compelling evidence to construct explanatory mediation effect models.

There are also some limitations to consider. First, while we concentrated on the most prevalent and modifiable metabolic risk factors as potential mediators to enhance clinical practice, the mediating function linking lifestyle and migraine remained incompletely explicated in this investigation. It is worth noting that the MVPA in this study did not completely differentiate between the two different types of aerobic exercise and strength training, which may affect the analysis and interpretation of the effects of specific exercise interventions. Future studies should further refine the measures of exercise, as well as explore the individual effects of aerobic exercise and strength training and their interactions, to provide more targeted exercise treatment programs. Furthermore, there may be interdependent mediating effects between different aspects of lifestyle. For example, prolonged LST is usually accompanied by negative mental health and behavioral changes that could potentially increase the likelihood of depression or the impact of coffee intake, while MVPA has a contrasting effect on both. It is challenging to distinguish between mediating and multiple effects in our MVMR outcomes. Therefore, these findings could also partially clarify the augmented risk of migraine. Secondly, the limitations of this study need to be considered as it only included participants of European ancestry, therefore caution should be taken before generalizing the findings to other ethnic groups with diverse lifestyles and cultural backgrounds. Third, an individual’s likelihood to report a phenotype to a physician can influence many phenotypes. For example, some of the migraine cases in this study were self-reported, which implies that more research is necessary to establish the generalizability of our findings to a broader spectrum of migraine symptoms.

## Conclusions

In conclusion, the present MR study offers genetic evidence supporting the detrimental impact of lifestyle LST on migraine and the beneficial impact of MVPA on migraine. Additionally, the study identifies hypertension as a mediator in the relationship between LST, MVPA, and migraine. The study provides causal evidence for the understanding of migraine etiology and offers prevention and intervention targets to reduce migraine prevalence and related disease burden.

### Electronic supplementary material

Below is the link to the electronic supplementary material.

Supplementary Material 1

Supplementary Material 2

Supplementary Material 3

Supplementary Material 4

Supplementary Material 5

Supplementary Material 6

Supplementary Material 7

Supplementary Material 8

Supplementary Material 9

## Data Availability

No datasets were generated or analysed during the current study.

## Abbreviations

GWAS: Genome-Wide Association Study
UVMR: Univariable Mendelian Randomization
MVMR: Multivariable Mendelian Randomization
SD: Standard Deviation
LST: Leisure Screen Time
MVPA: Moderate or/and Vigorous Physical Activity
DBP: Diastolic Blood Pressure
CSD: Cortical Spreading Depression
CGRP: Calcitonin-Related Peptide
MR: Mendelian Randomization
MVMR: Multivariate Mendelian Randomization
TSMR: Two-Sample Mendelian Randomization
PA: Physical Activity
SDKP: Sleep Disorder Knowledge Portal
GSCAN: GWAS & Alcohol and Nicotine Sequencing Consortium
UKB: UK Biobank
SESA: Sensitivity to Environmental Stress and Adversity
EPQ-RS: Eysenck Personality Questionnaire-Revised Short Form
GPC: Genetics of Personality Consortium
PGC: Psychiatric Genetics Consortium
NEO-FFI: NEO Five-Factor Personality Inventory
ICD-9: The International Classification of Diseases-9th classification
ICD-10: International Classification of Diseases-10th
DSM: The Diagnostic and Statistical Manual of Mental Disorders
BFI: Big Five Inventory
BMI: Body Mass Index
BF%: Body Fat Percentage
TG: Total Triglyceride
TC: Total Cholesterol
HDL-C: High-Density Lipoprotein – Cholesterol
LDL-C: Low-Density Lipoprotein – Cholesterol
SBP: Systolic Blood Pressure
PP: Pulse Pressure
PCs: Principal Components
GERA: Genetic Epidemiology Research in Adult Health and Aging
IHGC: International Headache Genetics Consortium
SNPs: Single Nucleotide Polymorphisms
IVs: Instrumental Variables
LD: Linkage Disequilibrium
IVW: Inverse Variance Weighted
MR-PRESSO: MR-Pleiotropy RESidual Sum and Outlier
BDNF: Brain-Derived Neurotrophic Factor
IGF-1: Insulin-like Growth Factor-1
VEGF: Vascular Endothelial Growth Factor
eNOS: endothelial Nitric Oxide Synthase

## References

[1] Global regional (2017) National incidence, prevalence, and years lived with disability for 328 diseases and injuries for 195 countries, 1990–2016: a systematic analysis for the global burden of Disease Study 2016. Lancet 390(10100):1211–1259. 10.1016/s0140-6736(17)32154-2 28919117 10.1016/s0140-6736(17)32154-2 PMC5605509

[2] Steiner TJ, Stovner LJ, Jensen R, Uluduz D, Katsarava Z (2020) Migraine remains second among the world’s causes of disability, and first among young women: findings from GBD2019. J Headache Pain 21(1):137. 10.1186/s10194-020-01208-0 33267788 10.1186/s10194-020-01208-0 PMC7708887

[3] Song TJ, Yun CH, Cho SJ, Kim WJ, Yang KI, Chu MK (2018) Short sleep duration and poor sleep quality among migraineurs: a population-based study. Cephalalgia 38(5):855–864. 10.1177/0333102417716936 28641451 10.1177/0333102417716936

[4] Peres MFP, Mercante JPP, Tobo PR, Kamei H, Bigal ME (2017) Anxiety and depression symptoms and migraine: a symptom-based approach research. J Headache Pain 18(1):37. 10.1186/s10194-017-0742-1 28324317 10.1186/s10194-017-0742-1 PMC5360747

[5] Aamodt AH, Stovner LJ, Hagen K, Bråthen G, Zwart J (2006) Headache prevalence related to smoking and alcohol use. The Head-HUNT Study. Eur J Neurol 13(11):1233–1238. 10.1111/j.1468-1331.2006.01492.x 17038038 10.1111/j.1468-1331.2006.01492.x

[6] Varkey E, Hagen K, Zwart JA, Linde M (2008) Physical activity and headache: results from the Nord-Trøndelag Health Study (HUNT). Cephalalgia: Int J Headache 28(12):1292–1297. 10.1111/j.1468-2982.2008.01678.x 10.1111/j.1468-2982.2008.01678.x 18771495

[7] Seng E, Martin P, Houle T (2022) Lifestyle factors and migraine. Lancet Neurol 21(10):911–921. 10.1016/s1474-4422(22)00211-3 36115363 10.1016/s1474-4422(22)00211-3

[8] Faisal Mohammad A, Stavroula A, Carlo B, Ewa KC-C, Daponte DA, Davide DL, Cherilyn F, Konstantinos K, Giorgos K, Mark B et al (2018) The association between migraine and physical exercise. J Headache Pain 19(1). 10.1186/s10194-018-0902-y 10.1186/s10194-018-0902-y PMC6134860 30203180

[9] Farris S, Thomas J, Abrantes A, Lipton R, Pavlovic J, Smitherman T, Irby M, Penzien D, Roth J, O’Leary K et al (2018) Pain worsening with physical activity during migraine attacks in women with overweight/obesity: a prospective evaluation of frequency, consistency, and correlates. Cephalalgia: Int J Headache 38(11):1707–1715. 10.1177/0333102417747231 10.1177/0333102417747231 PMC6373762 29237284

[10] Jason CO, Spencer CD, Hannah LT, Margaret P, Helen JB, Megan RC, Jeanetta CR, Todd AS, Colin AE, Alex LJ et al (2022) A Micro-longitudinal Study of naps, Sleep Disturbance, and Headache Severity in women with chronic migraine. Behav Sleep Med 21(2). 10.1080/15402002.2022.2050723

[11] Elizabeth KS, Cynthia DS (2016) Understanding migraine and psychiatric comorbidity. Curr Opin Neurol 29(3). 10.1097/wco.0000000000000309 10.1097/WCO.0000000000000309 26886355

[12] Cindy T, Alessandro V, Anton F, Tamara F, Annalisa G, Tatyana G, Irina Anna H, Yelena M, Lucas Hendrik O, Serena P et al (2020) Migraine and sleep disorders: a systematic review. J Headache Pain 21(1). 10.1186/s10194-020-01192-5 10.1186/s10194-020-01192-5 PMC7590682 33109076

[13] Nada Ahmad H, Niushen Z, Mallory F, Pixy B, Louise L, Sheena KA (2020) The role of Diet and Nutrition in Migraine triggers and treatment: a systematic literature review. Headache 60(7). 10.1111/head.13836 10.1111/head.13836 PMC7496357 32449944

[14] Alessandro P (2008) Alcohol and migraine: trigger factor, consumption, mechanisms. A review. J Headache Pain 9(1). 10.1007/s10194-008-0006-1 10.1007/s10194-008-0006-1 PMC3476173 18231712

[15] Karl BA, Anna PA (2019) Caffeine and primary (migraine) headaches-friend or foe? Front Neurol 10(0). 10.3389/fneur.2019.01275 10.3389/fneur.2019.01275 PMC6901704 31849829

[16] Andrea HW, Elizabeth KS (2023) The relationship of Tobacco Use and Migraine: a narrative review. Curr Pain Headache Rep 27(4). 10.1007/s11916-023-01103-8 10.1007/s11916-023-01103-8 PMC10006570 36905552

[17] Mi Ji L, Hyun Ah C, Hanna C, Chin-Sang C (2016) Caffeine discontinuation improves acute migraine treatment: a prospective clinic-based study. J Headache Pain 17(1). 10.1186/s10194-016-0662-5 10.1186/s10194-016-0662-5 PMC4975726 27492448

[18] Soomi C, Kyung Min K, Min Kyung C (2024) Coffee consumption and migraine: a population-based study. Sci Rep 14(1). 10.1038/s41598-024-56728-5 10.1038/s41598-024-56728-5 PMC10933282 38472388

[19] Md Rafiqul I, Dale RN (2023) Cross-trait analyses identify shared genetics between migraine, headache, and glycemic traits, and a causal relationship with fasting proinsulin. Hum Genet 142(8). 10.1007/s00439-023-02532-6 10.1007/s00439-023-02532-6 PMC10449981 36808568

[20] Rist P, Winter A, Buring J, Sesso H, Kurth T Migraine and the risk of incident hypertension among women. Cephalalgia: Int J Headache 2018, 38(12):1817–1824. 10.1177/0333102418756865 10.1177/0333102418756865 PMC6026578 29388437

[21] Elio A, Angelo A (2008) (0) Migraine and hypertension. *Neurol Sci*10.1007/s10072-008-0883-8

[22] Hagen K, Stovner LJ, Vatten L, Holmen J, Zwart JA, Bovim G (2002) Blood pressure and risk of headache: a prospective study of 22 685 adults in Norway. J Neurol Neurosurg Psychiatry 72(4):463–466. 10.1136/jnnp.72.4.463 11909904 10.1136/jnnp.72.4.463 PMC1737809

[23] C F F IH, J-A Z, B S W ML (2014) Blood pressure as a risk factor for headache and migraine: a prospective population-based study. Eur J Neurol 22(1). 10.1111/ene.12547 10.1111/ene.12547 25155744

[24] Yanjun G, Pamela MR, Iyas D, Franco G, Tobias K, Daniel IC (2020) A genome-wide cross-phenotype meta-analysis of the association of blood pressure with migraine. Nat Commun 11(1). 10.1038/s41467-020-17002-0 10.1038/s41467-020-17002-0 PMC7338361 32632093

[25] Ditte Georgina Z, Faisal Mohammad A, Song G, Mark BV, Anders H, Messoud A (2020) Plasma glucose levels increase during spontaneous attacks of Migraine with and without aura. Headache 60(4). 10.1111/head.13760 10.1111/head.13760 32031249

[26] Zeynep Oşar S, Derya U, Fatma Ela K, Feyza E, Huriye B, Uğur U, Sabahattin S, Baki G, Aksel S (2017) Determinants of glucose metabolism and the role of NPY in the progression of insulin resistance in chronic migraine. Cephalalgia 38(11). 10.1177/0333102417748928 10.1177/0333102417748928 29260593

[27] Ulrike H, Inna S, Katharina E-H, Cenk A (2012) Glucose modulation of spreading depression susceptibility. J Cereb Blood Flow Metab 33(2). 10.1038/jcbfm.2012.132 10.1038/jcbfm.2012.132 PMC3564186 22968322

[28] Balázs M, Barna P, Angelika V, Kitti P, Attila B, József N, Zoltán S, Gábor J, Mária D (2016) Diet-induced obesity alters dural CGRP release and potentiates TRPA1-mediated trigeminovascular responses. Cephalalgia 37(6). 10.1177/0333102416654883 10.1177/0333102416654883 27301459

[29] Ida Marchen Egerod CSJW, Rigmor Højland I, Sajedeh J (2021) Understanding the link between obesity and headache- with focus on migraine and idiopathic intracranial hypertension. J Headache Pain 22(1). 10.1186/s10194-021-01337-0 10.1186/s10194-021-01337-0 PMC8504002 34629054

[30] Olivia G, Matilde S, Gisela T, Gareth GL, Susan PM, Alexandra JS (2022) Alterations in metabolic flux in migraine and the translational relevance. J Headache Pain 23(1). 10.1186/s10194-022-01494-w 10.1186/s10194-022-01494-w PMC9523955 36175833

[31] Munvar Miya S, Siew Hua G (2015) Vitamin supplementation as possible prophylactic treatment against migraine with aura and menstrual migraine. Biomed Res Int 2015(0). 10.1155/2015/469529 10.1155/2015/469529 PMC4359851 25815319

[32] Elyas N-E, Mahmood Alizadeh S, Monireh D, Faezeh G, Abed G, Pishva A, Ali T-E (2018) The role of nutrients in the pathogenesis and treatment of migraine headaches: review. Biomed Pharmacother 102(0). 10.1016/j.biopha.2018.03.059 10.1016/j.biopha.2018.03.059 29571016

[33] Pamela MR, Julie EB, Nancy RC, JoAnn EM, Tobias K (2021) Effect of vitamin D and/or Marine n-3 fatty acid supplementation on changes in Migraine frequency and severity. Am J Med 134(6). 10.1016/j.amjmed.2020.11.023 10.1016/j.amjmed.2020.11.023 PMC8164960 33444588

[34] Domenico P, Guido P, Carlo M, Sara L, Serenella S, Nicola DS (2023) Vitamin D in neurological diseases. Int J Mol Sci 24(1). 10.3390/ijms24010087

[35] Smith GD, Ebrahim S (2003) Mendelian randomization’: can genetic epidemiology contribute to understanding environmental determinants of disease? Int J Epidemiol 32(1):1–22. 10.1093/ije/dyg070 12689998 10.1093/ije/dyg070

[36] Sanderson E (2021) Multivariable mendelian randomization and mediation. Cold Spring Harbor Perspect Med 11(2). 10.1101/cshperspect.a038984 10.1101/cshperspect.a038984 PMC7849347 32341063

[37] Wang Z, Emmerich A, Pillon N, Moore T, Hemerich D, Cornelis M, Mazzaferro E, Broos S, Ahluwalia T, Bartz T et al (2022) Genome-wide association analyses of physical activity and sedentary behavior provide insights into underlying mechanisms and roles in disease prevention. Nat Genet 54(9):1332–1344. 10.1038/s41588-022-01165-1 36071172 10.1038/s41588-022-01165-1 PMC9470530

[38] Jones SE, Lane JM, Wood AR, van Hees VT, Tyrrell J, Beaumont RN, Jeffries AR, Dashti HS, Hillsdon M, Ruth KS et al (2019) Genome-wide association analyses of chronotype in 697,828 individuals provides insights into circadian rhythms. Nat Commun 10(1):343. 10.1038/s41467-018-08259-7 30696823 10.1038/s41467-018-08259-7 PMC6351539

[39] Dashti H, Jones S, Wood A, Lane J, van Hees V, Wang H, Rhodes J, Song Y, Patel K, Anderson S et al (2019) Genome-wide association study identifies genetic loci for self-reported habitual sleep duration supported by accelerometer-derived estimates. Nat Commun 10(1):1100. 10.1038/s41467-019-08917-4 30846698 10.1038/s41467-019-08917-4 PMC6405943

[40] Lane J, Jones S, Dashti H, Wood A, Aragam K, van Hees V, Strand L, Winsvold B, Wang H, Bowden J et al (2019) Biological and clinical insights from genetics of insomnia symptoms. Nat Genet 51(3):387–393. 10.1038/s41588-019-0361-7 30804566 10.1038/s41588-019-0361-7 PMC6415688

[41] Liu M, Jiang Y, Wedow R, Li Y, Brazel D, Chen F, Datta G, Davila-Velderrain J, McGuire D, Tian C et al (2019) Association studies of up to 1.2 million individuals yield new insights into the genetic etiology of tobacco and alcohol use. Nat Genet 51(2):237–244. 10.1038/s41588-018-0307-5 30643251 10.1038/s41588-018-0307-5 PMC6358542

[42] Zhong V, Kuang A, Danning R, Kraft P, van Dam R, Chasman D, Cornelis M (2019) A genome-wide association study of bitter and sweet beverage consumption. Hum Mol Genet 28(14):2449–2457. 10.1093/hmg/ddz061 31046077 10.1093/hmg/ddz061 PMC6606847

[43] Nagel M, Speed D, van der Sluis S, Østergaard S (2020) Genome-wide association study of the sensitivity to environmental stress and adversity neuroticism cluster. Acta Psychiatrica Scandinavica 141(5):476–478. 10.1111/acps.13155 31972866 10.1111/acps.13155

[44] Nagel M, Jansen P, Stringer S, Watanabe K, de Leeuw C, Bryois J, Savage J, Hammerschlag A, Skene N, Muñoz-Manchado A et al (2018) Meta-analysis of genome-wide association studies for neuroticism in 449,484 individuals identifies novel genetic loci and pathways. Nat Genet 50(7):920–927. 10.1038/s41588-018-0151-7 29942085 10.1038/s41588-018-0151-7

[45] Eysenck SBG, Eysenck HJ, Barrett P (1985) A revised version of the psychoticism scale. Pers Indiv Differ 6(1):21–29. 10.1016/0191-8869(85)90026-1 10.1016/0191-8869(85)90026-1

[46] Costa PT, Mccrae RR (1992) Revised NEO personality inventory (NEO PI-R) and NEO five-factor inventory (NEO-FFI). Springer New York

[47] Chen Y, Lu T, Pettersson-Kymmer U, Stewart I, Butler-Laporte G, Nakanishi T, Cerani A, Liang K, Yoshiji S, Willett J et al (2023) Genomic atlas of the plasma metabolome prioritizes metabolites implicated in human diseases. Nat Genet 55(1):44–53. 10.1038/s41588-022-01270-1 36635386 10.1038/s41588-022-01270-1 PMC7614162

[48] Dennis J, Sealock J, Straub P, Lee Y, Hucks D, Actkins K, Faucon A, Feng Y, Ge T, Goleva S et al (2021) Clinical laboratory test-wide association scan of polygenic scores identifies biomarkers of complex disease. Genome Med 13(1):6. 10.1186/s13073-020-00820-8 33441150 10.1186/s13073-020-00820-8 PMC7807864

[49] Shin S, Fauman E, Petersen A, Krumsiek J, Santos R, Huang J, Arnold M, Erte I, Forgetta V, Yang T et al (2014) An atlas of genetic influences on human blood metabolites. Nat Genet 46(6):543–550. 10.1038/ng.2982 24816252 10.1038/ng.2982 PMC4064254

[50] Revez J, Lin T, Qiao Z, Xue A, Holtz Y, Zhu Z, Zeng J, Wang H, Sidorenko J, Kemper K et al (2020) Genome-wide association study identifies 143 loci associated with 25 hydroxyvitamin D concentration. Nat Commun 11(1):1647. 10.1038/s41467-020-15421-7 32242144 10.1038/s41467-020-15421-7 PMC7118120

[51] Yengo L, Sidorenko J, Kemper K, Zheng Z, Wood A, Weedon M, Frayling T, Hirschhorn J, Yang J, Visscher P (2018) Meta-analysis of genome-wide association studies for height and body mass index in ∼700000 individuals of European ancestry. Hum Mol Genet 27(20):3641–3649. 10.1093/hmg/ddy271 30124842 10.1093/hmg/ddy271 PMC6488973

[52] Wang H, Zhang F, Zeng J, Wu Y, Kemper K, Xue A, Zhang M, Powell J, Goddard M, Wray N et al (2019) Genotype-by-environment interactions inferred from genetic effects on phenotypic variability in the UK Biobank. Sci Adv 5(8):eaaw3538. 10.1126/sciadv.aaw3538 31453325 10.1126/sciadv.aaw3538 PMC6693916

[53] Willer C, Schmidt E, Sengupta S, Peloso G, Gustafsson S, Kanoni S, Ganna A, Chen J, Buchkovich M, Mora S et al (2013) Discovery and refinement of loci associated with lipid levels. Nat Genet 45(11):1274–1283. 10.1038/ng.2797 24097068 10.1038/ng.2797 PMC3838666

[54] Manning A, Hivert M, Scott R, Grimsby J, Bouatia-Naji N, Chen H, Rybin D, Liu C, Bielak L, Prokopenko I et al (2012) A genome-wide approach accounting for body mass index identifies genetic variants influencing fasting glycemic traits and insulin resistance. Nat Genet 44(6):659–669. 10.1038/ng.2274 22581228 10.1038/ng.2274 PMC3613127

[55] Jiang L, Zheng Z, Fang H, Yang J (2021) A generalized linear mixed model association tool for biobank-scale data. Nat Genet 53(11):1616–1621. 10.1038/s41588-021-00954-4 34737426 10.1038/s41588-021-00954-4

[56] Evangelou E, Warren H, Mosen-Ansorena D, Mifsud B, Pazoki R, Gao H, Ntritsos G, Dimou N, Cabrera C, Karaman I et al (2018) Genetic analysis of over 1 million people identifies 535 new loci associated with blood pressure traits. Nat Genet 50(10):1412–1425. 10.1038/s41588-018-0205-x 30224653 10.1038/s41588-018-0205-x PMC6284793

[57] Kurki MI, Karjalainen J, Palta P, Sipilä TP, Kristiansson K, Donner KM, Reeve MP, Laivuori H, Aavikko M, Kaunisto MA et al (2023) FinnGen provides genetic insights from a well-phenotyped isolated population. Nature 613(7944):508–518. 10.1038/s41586-022-05473-8 36653562 10.1038/s41586-022-05473-8 PMC9849126

[58] Choquet H, Yin J, Jacobson A, Horton B, Hoffmann T, Jorgenson E, Avins A, Pressman A (2021) New and sex-specific migraine susceptibility loci identified from a multiethnic genome-wide meta-analysis. Commun Biology 4(1):864. 10.1038/s42003-021-02356-y 10.1038/s42003-021-02356-y PMC8298472 34294844

[59] Purcell S, Neale B, Todd-Brown K, Thomas L, Ferreira M, Bender D, Maller J, Sklar P, de Bakker P, Daly M et al (2007) PLINK: a tool set for whole-genome association and population-based linkage analyses. Am J Hum Genet 81(3):559–575. 10.1086/519795 17701901 10.1086/519795 PMC1950838

[60] Bowden J, Holmes MV (2019) Meta-analysis and mendelian randomization: a review. Res Synthesis Methods 10(4):486–496. 10.1002/jrsm.1346 10.1002/jrsm.1346 PMC6973275 30861319

[61] Global regional (2018) National burden of migraine and tension-type headache, 1990–2016: a systematic analysis for the global burden of Disease Study 2016. Lancet Neurol 17(11):954–976. 10.1016/s1474-4422(18)30322-3 30353868 10.1016/s1474-4422(18)30322-3 PMC6191530

[62] Krøll LS, Hammarlund CS, Linde M, Gard G, Jensen RH (2018) The effects of aerobic exercise for persons with migraine and co-existing tension-type headache and neck pain. A randomized, controlled, clinical trial. Cephalalgia: Int J Headache 38(12):1805–1816. 10.1177/0333102417752119 10.1177/0333102417752119 29333870

[63] Malkki H (2016) Migraine. Long screen time exposure could increase the risk of migraine. Nat Reviews Neurol 12(1):4. 10.1038/nrneurol.2015.238 10.1038/nrneurol.2015.238 26678985

[64] Claudia HO, Stephanie D, Marie CE, Wolf-Dieter G, Melanie G, Armin K, Uwe N, Henrik S, Michael S, Burkhard W (2014) Does an aerobic endurance programme have an influence on information processing in migraineurs? J Headache Pain 15(1). 10.1186/1129-2377-15-11 10.1186/1129-2377-15-11 PMC4017768 24528557

[65] Yohannes WW, Arão BDO (2022) What is the efficacy of aerobic exercise versus strength training in the treatment of migraine? A systematic review and network meta-analysis of clinical trials. J Headache Pain 23(1). 10.1186/s10194-022-01503-y 10.1186/s10194-022-01503-y PMC9563744 36229774

[66] Johannes F, Jörg S, Laura B, Matthias KA, Hartmut K, Beat L, Peter G (2015) A runner’s high depends on cannabinoid receptors in mice. Proc Natl Acad Sci U S A 112(42). 10.1073/pnas.1514996112 10.1073/pnas.1514996112 PMC4620874 26438875

[67] Eduardo MMP, Thais C, Renato SM-J, Thiago TG, Ercole dCR, Eduardo L, Charlene B, Andrea CD (2013) Neuroscience of exercise: from neurobiology mechanisms to mental health. Neuropsychobiology 68(1). 10.1159/000350946 10.1159/000350946 23774826

[68] Marinei LP, Rafael AM, Daniel PK, Salvador GN, Bruna E, Hirofumi T, Alexandre ML (2020) Different exercise training modalities produce similar endothelial function improvements in individuals with prehypertension or hypertension: a randomized clinical trial Exercise, endothelium and blood pressure. Sci Rep 10(1). 10.1038/s41598-020-64365-x 10.1038/s41598-020-64365-x PMC7203179 32376984

[69] Ylva H, Michael N (2016) Cardiovascular adaptations to Exercise Training. Compr Physiol 6(1). 10.1002/cphy.c140080 10.1002/cphy.c140080 26756625

[70] Guo Y, Rist P, Daghlas I, Giulianini F, Kurth T, Chasman D (2020) A genome-wide cross-phenotype meta-analysis of the association of blood pressure with migraine. Nat Commun 11(1):3368. 10.1038/s41467-020-17002-0 32632093 10.1038/s41467-020-17002-0 PMC7338361

[71] Kazuki H, Bradley JB, Bahram A, Payal G, Bei C, Rachael B, Joshua JM, Marcus LE, Patrick M, Daniel K et al (2018) Daily muscle stretching enhances blood flow, endothelial function, capillarity, vascular volume and connectivity in aged skeletal muscle. J Physiol 596(10). 10.1113/jp275459 10.1113/JP275459 PMC5978284 29623692

[72] Sachin AG, Michael SW (2012) Relationships between vascular oxygen sensing mechanisms and hypertensive disease processes. Hypertension 60(2). 10.1161/hypertensionaha.112.190702 10.1161/HYPERTENSIONAHA.112.190702 PMC3401320 22710643

[73] Gretchen ET, Jagdish K (2014) Vascular biomarkers in migraine. Cephalalgia 35(2). 10.1177/0333102414544976 10.1177/0333102414544976 25281220

[74] Montagni I, Guichard E, Carpenet C, Tzourio C, Kurth T (2016) Screen time exposure and reporting of headaches in young adults: a cross-sectional study. Cephalalgia: Int J Headache 36(11):1020–1027. 10.1177/0333102415620286 10.1177/0333102415620286 26634831

[75] Josefine L, Amalie B-U, Merve C, Nanette Marinette Monique D (2021) Headache in children and adolescents: the association between screen time and headache within a clinical Headache Population. Neuropediatrics 53(4). 10.1055/s-0041-1740550 10.1055/s-0041-1740550 34905787

[76] Ilaria M, Elie G, Claire C, Christophe T, Tobias K (2015) Screen time exposure and reporting of headaches in young adults: a cross-sectional study. Cephalalgia 36(11). 10.1177/0333102415620286 10.1177/0333102415620286 26634831

[77] Chantal AV, Katrina T, Megan CN (2020) Associations of leisure screen time with cardiometabolic biomarkers in college-aged adults. J Behav Med 43(6). 10.1007/s10865-020-00161-2 10.1007/s10865-020-00161-2 PMC7677220 32451650

[78] Josephine YC, Anne G, Kristian M, Jostein H, Turid LH, Adrian EB, Hidde P, vdP (2013) Cross-sectional associations of total sitting and leisure screen time with cardiometabolic risk in adults. Results from the HUNT study, Norway. J Sci Med Sport 17(1). 10.1016/j.jsams.2013.03.004 10.1016/j.jsams.2013.03.004 23619159

[79] Amit S, Michael JM (2012) Metabolic syndrome and migraine. Front Neurol 3(0). 10.3389/fneur.2012.00161 10.3389/fneur.2012.00161 PMC3500825 23181051

[80] Sanjeev KB, Jayantee K, Usha KM (2012) Metabolic syndrome and insulin resistance in migraine. J Headache Pain 13(4). 10.1007/s10194-012-0416-y 10.1007/s10194-012-0416-y PMC3356472 22278639

[81] Kristina B, Erik L, Johan S, Peter MN, Sölve E, Nancy LP, Lars L (2018) Interaction between physical activity and television time on blood pressure level: cross-sectional data from 45 000 individuals. J Hypertens 36(5). 10.1097/hjh.0000000000001675 10.1097/HJH.0000000000001675 29369146

[82] Ornello R, Ripa P, Pistoia F, Degan D, Tiseo C, Carolei A, Sacco S (2015) Migraine and body mass index categories: a systematic review and meta-analysis of observational studies. J Headache Pain 16:27. 10.1186/s10194-015-0510-z 25903159 10.1186/s10194-015-0510-z PMC4385329

[83] Ghorbani Z, Togha M, Rafiee P, Ahmadi ZS, Rasekh Magham R, Djalali M, Shahemi S, Martami F, Zareei M, Razeghi Jahromi S et al (2020) Vitamin D3 might improve headache characteristics and protect against inflammation in migraine: a randomized clinical trial. Neurol Sci 41(5):1183–1192. 10.1007/s10072-019-04220-8 31897949 10.1007/s10072-019-04220-8

