# Supplementary material for: The role of hypertension in the relationship between leisure screen time, physical activity and migraine: a 2-sample Mendelian randomization study
Source: J Headache Pain. 2024 Jul 24;25(1):122. doi: 10.1186/s10194-024-01820-4 (PMC11267787; doi:10.1186/s10194-024-01820-4)
Supplement: Supplementary file 1 — Supplementary Material 1 [file 10194_2024_1820_MOESM1_ESM.docx]

Table S1. Summary of the GWAS data of studied lifestyle factors.

| **Phenotype** | **Sample size** | **Author** | **Unit** | **Consortium/cohort** | **PubMed ID** | **Year of publication** | **Adjustment** |
| --- | --- | --- | --- | --- | --- | --- | --- |
| **Physical activity** | | | | | | |  |
| MVPA | 608,595 | Wang et al | MVPA activer compared to inactiver | Meta | 36071172 | 2022 | Age, age-squared, principal components reflecting population structure and additional study-specific covariates |
| **Sedentary behaviors** | | | | | | |  |
| Sedentary behavior at work | 372,609 | Wang et al | Individuals who reported sedentary at work (yes vs. no) | Meta | 36071172 | 2022 | Age, age-squared, principal components reflecting population structure and additional study-specific covariates |
| Sedentary commuting | 159,606 | Wang et al | Individuals who reported sedentary commuting (yes vs. no) | Meta | 36071172 | 2022 |  |
| Leisure screen time | 526,725 | Wang et al | 1 SD increase in hour of leisure screen per day | Meta | 36071172 | 2022 |  |
| **Sleep disturbances** | | | | | |  |  |
| Chronotype | 449,734 | Jones et al | Morning' person compared with 'Night' person | SDKP | 30696823 | 2019 | Age, sex, study centre and a derived variable representing genotyping release |
| Sleep duration | 446,118 | Dashti et al | 1 SD increase in an hour of sleep duration | SDKP | 30846698 | 2019 | Age, sex, 10 principal components of ancestry, genotyping array, and genetic correlation matrix |
| Long sleep duration | 339,926 | Dashti et al | Long duration (≥9 h) compared with normal duration (7–8 h) | SDKP | 30846698 | 2019 |  |
| Short sleep duration | 411,934 | Dashti et al | Short duration (<7 h) compared with normal duration (7–8 h) | SDKP | 30846698 | 2019 |  |
| Insomnia | 386,533 | Lane et al | Insomnia individuals compared to non-insomnia individual | SDKP | 30804566 | 2019 |  |
| **Poor dietary habits** | | | | | |  |  |
| Age of smoking initiation | 341,427 | Liu et al | SD in the prevalence of smoking initiation | GSCAN | 30643251 | 2019 | Age, sex, and the first ten genetic principal  components |
| Smoking status | 1,232,091 | Liu et al | Ever smoked regularly compared with never smoked | GSCAN | 30643251 | 2019 |  |
| Cigarettes consumption | 337,334 | Liu et al | 1 SD increase in number of cigarettes smoked per day | GSCAN | 30643251 | 2019 |  |
| Smoking cessation | 547,219 | Liu et al | Current smokers compared with former smokers | GSCAN | 30643251 | 2019 |  |
| Alcohol consumption | 941,280 | Liu et al | 1 SD increase of log-transformed alcoholic drinks/wk | GSCAN | 30643251 | 2019 |  |
| Coffee intake | 375,833 | Zhong et al | 50% increase (an increase from 1 cup to 1.5 cups). | UKB | 31046077 | 2019 | Age, sex, BMI, total energy, proportion of 24 h recalls self-reported |
| **Affective disorders** | | | | | |  |  |
| SESA | 351,827 | Nagel et al | Answer to three SESA questions (yes vs. no) | UKB | 31972866 | 2020 | Age, sex,  Townsend deprivation index, genotyping array, and  ten principal components |
| Neuroticism | 390,278 | Nagel et al | Answer to the 12 dichotomous items of EPQ-RS (yes or no) | Meta | 29942085 | 2018 | Age, sex, Townsend deprivation index, genotype array, and ten genetic European-based principal components |
| Depression | 381,455 | Nagel et al | Answer to two depression items (yes or no) | Meta | 29942085 | 2018 |  |
| Depressed affect subcluster | 357,957 | Nagel et al | Answer to the four EPQ-RS items (yes or no) | UKB | 29942085 | 2018 |  |
| Worry subcluster | 348,219 | Nagel et al | Answer to the other four EPQ-RS items (yes or no) | UKB | 29942085 | 2018 |  |
| **Migraine** | | | | | |  |  |
| Discovery | | | | | |  |  |
|  | 306,314 | Kurki et al | Event (Medical records) | FinnGen | 36653562 | 2023 | Age, sex, and genetic ancestry principal components |
| Replication | | | | | |  |  |
|  | 513,266 | Choquet et al | Event (Medical records or self-report from questionnaire) | Meta | 34294844 | 2021 | Age, sex, and genetic ancestry principal components |

Abbreviation: MVPA, Moderate-to-vigorous intensity physical activity during leisure time; SDKP, Sleep Disorder Knowledge Portal; GCSAN, GWAS & Sequencing Consortium of Alcohol and Nicotine; UKB, UK Biobank; SESA, Sensitivity to environmental stress and adversity; EPQ-RS, Eysenck Personality Questionnaire-Revised Short Form.
