# Supplementary material for: The role of hypertension in the relationship between leisure screen time, physical activity and migraine: a 2-sample Mendelian randomization study
Source: J Headache Pain. 2024 Jul 24;25(1):122. doi: 10.1186/s10194-024-01820-4 (PMC11267787; doi:10.1186/s10194-024-01820-4)
Supplement: Supplementary file 2 — Supplementary Material 2 [file 10194_2024_1820_MOESM2_ESM.docx]

Table S2. Epidemiological evidence for the relationship between the studied lifestyle factors and candidate mediators with migraine.

| **Genotype** | **Study design** | **Sample size** | **Data collection** | **Epidemiological evidence** |
| --- | --- | --- | --- | --- |
| **Lifestyle factors** | | | | |
| Physical activity[1] | Prospective cohort study | 1125 patients with EM | 90 days | Physical activity was associated with decreased migraine attack risk |
| Leisure screen time [2] | Cross-sectional study | 31,022 adolescents | NA | After adjusting for age, socioeconomic status, depressed mood, school-related stress, and physical activity, regression analyses showed that computer use, computer games, and TV viewing increased the risk of headaches. |
| Sleep disorder [3] | Retrospective cohort study | 133,262 subjects | 3 years | A survival analysis using a Cox proportional risk model with time-dependent covariates showed that sleep disorders were positively associated with the diagnosis of migraine (hazard ratio, 1.591). |
| Smoking and drink behavior [4] | Cross-sectional study | 51,383 subjects | NA | After adjusting for sex, age, and education, an unit of alcohol consumption associated with 20% decreasing prevalence of migraine (OR 0.8, 95%CI 0.7-0.9), and slightly higher  headache prevalence OR amongst never smokers exposed to passive smoking compared with those not  exposed to passive smoking (OR 1.4, 95%CI 1.1-1.6) |
| Coffee intake [5] | Prospective cohort study | 108 migraine patients who consumed caffeine drinks daily | 1-month | After controlling for chronic migraine, BMI, allodynia, depression, anxiety, antiemetic use, different triptans, and use of prophylactic medication, multivariable logistic regression model shown that caffeine abstinence was independently associated with an excellent efficacy of acute treatment (OR 3.2, 95%CI 1.2–8.4) |
| Affective disorders [6] | Cross-sectional study | 782 subjects | NA | The leading OR observed in the migraine patients relative to those without migraine were anxiety related, “Not being able to stop or control worrying” on a daily basis (OR 49.2, 95%CI 13.6–178.2), “trouble relaxing” (25.7, 7.1–92.6), “Feeling nervous, anxious or on edge” on a daily basis (25.4, 6.8–93.8), and “worrying too much about different things” (24.4, 7.6–77.6). Irritability had a significant increase in migraine risk (OR 3.8, 95%CI 1.9–7.8) |
| **Mediators** | | | | |
| Vitamins [7] | Cross-sectional study | 266 women | NA | After adjusting for age, physical activity, BMI, education, marital status and job, dietary pattern (vitamin B1, carbohydrate, vitamin B3, vitamin B9, protein and total fibre) was associated with the intensity of migraine (β 0.37; 95%CI, 0.13-0.61; *P*-value < 0.001), nutrient pattern (Ca, vitamin A, vitamin K, vitamin C, vitamin B6, vitamin B2 and Mg) was negatively related with migraine disability (β –3·14; 95 % CI, -5.47~-0.81; *P*-value = 0.01), and a significant positive association between vitamin D and B12 and headache duration (β1.89; 95 %CI;0.49-3.29, *P*-value = 0·008) |
| Obesity [8] | Retrospective cross-sectional study | 2,094,862 adolescents | NA | After adjusting for residential socioeconomic status, education status, and cognitive performance, the adjusted ORs for migraine were 1.11 (95%CI, 1.06-1.16), 1.13 (1.08-1.17), and 1.24 (1.19-1.30), for the underweight, overweight, and obesity subgroups, respectively, compared to the reference group of low-normal BMI (5th-49th percentile) among males, the respective adjusted ORs were 1.12 (1.05–1.19), 1.23 (1.19–1.28), and 1.38 (1.31–1.46) among females. |
| Lipid metabolism [9] | Longitudinal cross-sectional study | 1389 subjects | NA | After adjusting of age, gender, smoking status, ever cholesterol medication use, alcohol consumption, blood pressure, and BMI, a significant correlations between higher tertiles of total cholesterol and migraine with aura compared to participants without headache, with OR of 4.67 (0.99–21.97) for the 2nd tertile and 5.97 (1.29–27.61) for the 3rd tertile, and strong associations between triglycerides and migraine with aura, with an OR of 4.42 (1.32–14.77) for the 3rd tertile. |
| Glucose metabolism [10] | Prospective cohort study | 27 chronic migraineurs and 37 healthy controls | 24 years | After adjusting for age, level of education, family history of diabetes, BMI, smoking status, hypertension, physical activity, use of oral contraceptives, menopausal status, menopausal hormone therapy uses, and handedness, a lower risk of type 2 diabetes was observed for women with active migraine compared with women with no migraine history (HR 0.70, 95% CI 0.58-0.85). |
| Blood pressure [11] | Prospective cohort study | 13,852 | 11 years | After adjusting of age, BMI, education, HADS score,  smoking, physical activity, caffeine intake, cholesterol  levels and alcohol overuse, it was found that a high PP was associated with a lower odds ratio (OR 0.73, 95% CI 0.64-0.82) of migraine, and high SBP was associated with an odds ratio (OR 0.84, 95% CI 0.77-0.93) of migraine. However, no association was found between DBP and the risk of migraine. |

1. Casanova A, Vives-Mestres M, Donoghue S, Mian A, Wöber C: **The role of avoiding known triggers, embracing protectors, and adhering to healthy lifestyle recommendations in migraine prophylaxis: Insights from a prospective cohort of 1125 people with episodic migraine**. *Headache* 2023, **63**(1):51-61. <https://doi.org/10.1111/head.14451>

2. Torsheim T, Eriksson L, Schnohr CW, Hansen F, Bjarnason T, Välimaa R: **Screen-based activities and physical complaints among adolescents from the Nordic countries**. *BMC Public Health* 2010, **10**(1):324. <https://doi.org/10.1186/1471-2458-10-324>

3. Kim SJ, Han K-T, Jang S-Y, Yoo K-B, Kim SJ: **The Association between Migraine and Types of Sleep Disorder**. *International Journal of Environmental Research and Public Health* 2018, **15**(12):2648.

4. Aamodt AH, Stovner LJ, Hagen K, Bråthen G, Zwart J: **Headache prevalence related to smoking and alcohol use. The Head-HUNT Study**. *Eur J Neurol* 2006, **13**(11):1233-1238. <https://doi.org/10.1111/j.1468-1331.2006.01492.x>

5. Lee MJ, Choi HA, Choi H, Chung C-S: **Caffeine discontinuation improves acute migraine treatment: a prospective clinic-based study**. *The Journal of Headache and Pain* 2016, **17**(1):71. <https://doi.org/10.1186/s10194-016-0662-5>

6. Peres MFP, Mercante JPP, Tobo PR, Kamei H, Bigal ME: **Anxiety and depression symptoms and migraine: a symptom-based approach research**. *J Headache Pain* 2017, **18**(1):37. <https://doi.org/10.1186/s10194-017-0742-1>

7. Bahrampour N, Mirzababaei A, Yarizadeh H, Barekzai A, Khorsha F, Clark C, Mirzaei K: **The relationship between dietary nutrients patterns and intensity and duration of migraine headaches**. *The British journal of nutrition* 2022:1-8. <https://doi.org/10.1017/s0007114522000046>

8. Zloof Y, Tsur A, Simchoni M, Derazne E, Tzur D, Honig A, Braun M, Ganelin-Cohen E, Amarilyo G, Pinhas-Hamiel O *et al*: **Body mass index and migraine in adolescence: A nationwide study**. *Cephalalgia : an international journal of headache* 2023, **43**(10):3331024231209309. <https://doi.org/10.1177/03331024231209309>

9. Rist P, Tzourio C, Kurth T: **Associations between lipid levels and migraine: cross-sectional analysis in the epidemiology of vascular ageing study**. *Cephalalgia : an international journal of headache* 2011, **31**(14):1459-1465. <https://doi.org/10.1177/0333102411421682>

10. Fagherazzi G, El Fatouhi D, Fournier A, Gusto G, Mancini F, Balkau B, Boutron-Ruault M, Kurth T, Bonnet F: **Associations Between Migraine and Type 2 Diabetes in Women: Findings From the E3N Cohort Study**. *JAMA neurology* 2019, **76**(3):257-263. <https://doi.org/10.1001/jamaneurol.2018.3960>

11. Fagernæs C, Heuch I, Zwart J, Winsvold B, Linde M, Hagen K: **Blood pressure as a risk factor for headache and migraine: a prospective population-based study**. *European journal of neurology* 2015, **22**(1):156-162, e110-151. <https://doi.org/10.1111/ene.12547>
