# Supplementary material for: The role of hypertension in the relationship between leisure screen time, physical activity and migraine: a 2-sample Mendelian randomization study
Source: J Headache Pain. 2024 Jul 24;25(1):122. doi: 10.1186/s10194-024-01820-4 (PMC11267787; doi:10.1186/s10194-024-01820-4)
Supplement: Supplementary file 3 — Supplementary Material 3 [file 10194_2024_1820_MOESM3_ESM.docx]

Table S3. Summary of the GWAS data of studied mediators.

| **Phenotype** | **Sample size** | **Author** | **Unit** | **Consortium/cohort** | **PubMed ID** | **Year of publication** |
| --- | --- | --- | --- | --- | --- | --- |
| Retinol | 8,247 | Chen et al | SD (5.3 ug/L) | CLSA | 36635386 | 2013 |
| Vitamin B12 | 19,415 | Dennis et al | SD (1.0 pmol/L) | BioUV | 33441150 | 2021 |
| Vitamin C | 2,085 | Shin et al | SD (0.4 mg/L) | Meta | 24816252 | 2014 |
| 25-Hydroxyvitamin D | 417,580 | Revez et al | SD (3.0 nmol/L) | UKB | 32242144 | 2020 |
| alpha-tocopherol | 7,725 | Shin et al | SD (0.2 mg/L) | Meta | 24816252 | 2014 |
| gamma-tocopherol | 6,226 | Shin et al | SD (0.2 mg/L) | Meta | 24816252 | 2014 |
| Body mass index | 456,426 | Yengo et al | SD (4.7 kg/m^2^) | UKB | 30124842 | 2018 |
| BF% | 401,772 | Wang et al | SD (1.1 %) | UKB | 31453325 | 2019 |
| Triglycerides | 94,595 | Willer et al | SD (90.7 mg/dL) | GLGC | 24097068 | 2013 |
| Total cholesterol | 94,595 | Willer et al | SD (41.8 mg/dL) | GLGC | 24097068 | 2013 |
| LDL cholesterol | 94,595 | Willer et al | SD (38.7 mg/dL) | GLGC | 24097068 | 2013 |
| HDL cholesterol | 94,595 | Willer et al | SD (15.5 mg/dL) | GLGC | 24097068 | 2013 |
| Type 2 diabetes mellitus | 456,348 | Jiang et al | Event | UKB | 34737426 | 2021 |
| Fasting glucose | 58,074 | Manning et al | SD (0.5 mmol/L) | MAGIC | 22581228 | 2012 |
| Fasting insulin | 58,074 | Manning et al | SD (40.2 pmol/L) | MAGIC | 22581228 | 2012 |
| Hypertension | 455,303 | Jiang et al | Event | UKB | 34737426 | 2021 |
| SBP | 757,601 | Evangelou et al | SD (20.7 mmHg) | ICBP | 30224653 | 2018 |
| DBP | 757,601 | Evangelou et al | SD (11.3 mmHg) | ICBP | 30224653 | 2018 |
| PP | 757,601 | Evangelou et al | SD (14.2 mmHg) | ICBP | 30224653 | 2018 |

CLSA, The Canadian Longitudinal Study of Aging; GLGC, Global Lipids Genetics Consortium; MAGIC, Meta-Analyses of Glucose and Insulin-related traits Consortium; ICBP, International Consortium of Blood Pressure.
